# Sirtuin3 rs28365927 functional variant confers to the high risk of non-alcoholic fatty liver disease in Chinese Han population

**DOI:** 10.1186/s12944-021-01520-x

**Published:** 2021-08-26

**Authors:** Li-jie Chen, Jing Guo, Song-xia Zhang, Ying Xu, Qing Zhao, Wei Zhang, Jian Xiao, Yao Chen

**Affiliations:** 1 Department of Clinical Pharmacology, Xiangya Hospital, Central South University, Changsha, 410008 Hunan P. R. China; 2 Institute of Clinical Pharmacology, Central South University, Changsha, Hunan China; 3 Engineering Research Center of Applied Technology of Pharmacogenomics, Ministry of Education, Changsha, Hunan China; 4 National Clinical Research Center for Geriatric Disorders, Changsha, Hunan China; 5 Department of Pharmacy, Xiangya Hospital, Central South University, Changsha, Hunan China

**Keywords:** Sirtuin3, Non-alcoholic fatty liver disease, Functional mutation, rs28365927

## Abstract

**Background:**

Non-alcoholic fatty liver disease (NAFLD) is a multifactorial condition associated with aging, insulin resistance, metabolic syndrome, genetic factors and more. Although genetic traits are among the most important risks factors for NAFLD, the understanding of their influence is still quite limited. The present study aimed at identifying novel single nucleotide polymorphisms (SNPs) that may confer a risk for NAFLD in the Han Chinese population.

**Methods:**

Based on the “two-hit hypothesis”, candidate SNPs, including Sirtuin3 rs28365927, were genotyped by MassARRAY in B-type ultrasonography-proven NAFLD patients (*n* = 292) and healthy controls (*n* = 387).

**Results:**

In a model analysis of individuals matched based on gender and age that compared 223 NAFLD and 223 non-NAFLD patients, the rs28365927 GA + AA genotype was a significant risk factor for the development of NAFLD in a dominant model. Rs28365927 was significantly associated with a higher NAFLD risk in both an additive model (A vs G) and genotypic model (GA vs GG). Among the NAFLD patients, serum levels of total bilirubin (TBIL), DBIL direct bilirubin (DBIL) and glutamic-pyruvic transaminase (ALT) in rs28365927 A allele carriers (GA + AA) were 11.1, 14.7 and 41.5% higher, respectively, than in non-carriers (GG). Furthermore, among the NAFLD patients, the carriers of Rs28365927 allele A were positively correlated with higher ALT levels.

**Conclusion:**

Sirtuin3 rs28365927 functional variant confers to the high risk of non-alcoholic fatty liver disease in Chinese Han population. The rs28365927 A allele significantly increased the ALT levels of NAFLD patients.

**Supplementary Information:**

The online version contains supplementary material available at 10.1186/s12944-021-01520-x.

## Introduction

Non-alcoholic fatty liver disease (NAFLD) is a common cause of liver-related death that affects a quarter of adults worldwide, with the highest prevalence in the Middle East (31.8%) and South America (30.5%) and the lowest in Africa (13.5%). Furthermore, the prevalence of NAFLD in China is 27.4% [1]. This disease is characterized by an excess accumulation of fat (in the form of triglycerides) in the hepatocytes (> 5% fat content in the liver, referred to as steatosis), leading to non-alcoholic fatty liver [2]. NAFLD includes a broad range of pathologic features, ranging from benign and reversible simple steatosis to more severe non-alcoholic steatohepatitis (NASH), which can progress to fibrosis, cirrhosis and even hepatocellular carcinoma [3]. NAFLD-related diabetes, atherosclerosis and liver disease have significantly increased complications and mortality, posing a serious threat to life and health [4].

NAFLD is a multifactorial disease with obvious individual susceptibility, and a number of risk factors have been identified, such as metabolic syndrome, sedentary lifestyle, diet, aging, gender, genetic factors and other conditions [2, 5–11]. Genetic factors are among the most important determinants for individual susceptibility to NAFLD, and great progress has been made in identifying these factors in recent years. For example, rs738409, a functional loss variant of patatin-like phospholipase domain containing 3 (PNPLA3) (I148M), has been associated with the severity of steatosis and fibrosis as well as the presence of NASH [12]. The glucokinase regulator (GCKR) rs780094 (P446L) mutation increases the accumulation of liver fat by stimulating fat generation and glucose uptake [13]. In the Han Chinese population, sterol regulatory element binding transcription factor 2(SREBP-2) rs2228314 carriers of G (CG + GG) may be at an increased risk for NAFLD [14].

Although considerable progress has been made in the study of individual susceptibilities to NAFLD, much is still unknown. Therefore, identifying novel SNPs that may confer a risk for NAFLD still remains of great significance [15]. The purpose of the current study was to discover new SNPs that contribute to the individual susceptibility risk for NAFLD in the Han Chinese population.

## Subjects and methods

### Study subjects

The clinical trial was performed in accordance with the principles of the Declaration of Helsinki and its appendices. It was approved by the Medical Ethics Committee of the Department of Clinical Pharmacology, Xiangya Hospital, Central South University (Changsha, China) and was registered with the Chinese Clinical Trial Registry (no. ChiCTR-ROC-15006899). All participants provided written informed consent before participation.

This study recruited 292 NAFLD patients and 387 non-NAFLD patients. Participants were recruited from the Health Management Center of Xiangya Hospital between June 2015 and January 2020. The diagnosis of NAFLD was performed according to the guidelines for the diagnosis and treatment of NAFLD of China (2010), the European Association for the Study of the Liver and the American Association for the Study of Liver Diseases criteria [16]. The inclusion criteria were as follows: B-type ultrasonography of the liver showing normal sonography or hepatic imaging findings consistent with the diagnostic criteria for diffuse fatty liver without other explanations and serum glutamic-pyruvic transaminase (ALT) and/or glutamic oxaloacetic transaminase (AST) and gamma-glutamyltransferase (GGT) levels continuously raised for over half a year in patients with components related to metabolic syndrome with unknown causes. All subjects with other causes of liver disease were excluded, including excess alcohol intake (≥ 20 g/d), viral liver infection, hepatic cyst, liver haemangioma, drug-induced hepatitis, schistosomal liver, liver calcifications, cirrhosis and other liver diseases.

**Fig. 1:**
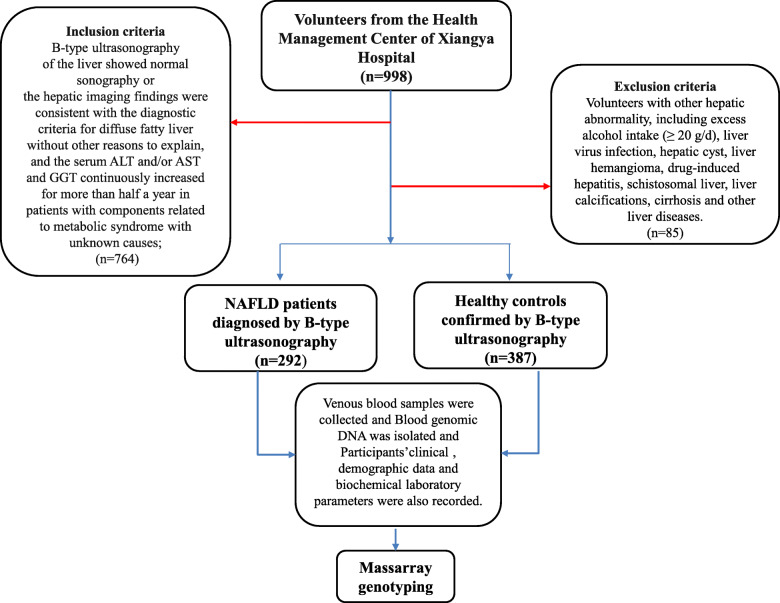
Diagram of the screening procedure for patients of NAFLD and non-NAFLD. Abbreviations: NAFLD, non-alcoholic fatty liver disease; ALT, glutamic-pyruvic transaminase; AST, glutamic oxalacetic transaminase; GGT, gamma-glutamyl transferase

Venous blood samples (3–5 mL/each) were collected from each patient for DNA extraction using disposable venous blood lancets and disposable blood collection tubes. Blood genomic DNA was isolated with a commercial DNA extraction kit (Omega Bio-Tek, GA, USA) according to the manufacturer’s instructions and stored at − 80 °C until use. The participants’ clinical and demographic data, including age, gender, body mass index (BMI), systolic blood pressure (SBP), diastolic blood pressure (DBP), waist circumference (WC), hip circumference (HC) and biochemical laboratory parameters, were also recorded (Fig. 1).

### Genetic variants selection and genotyping

**Fig. 2:**
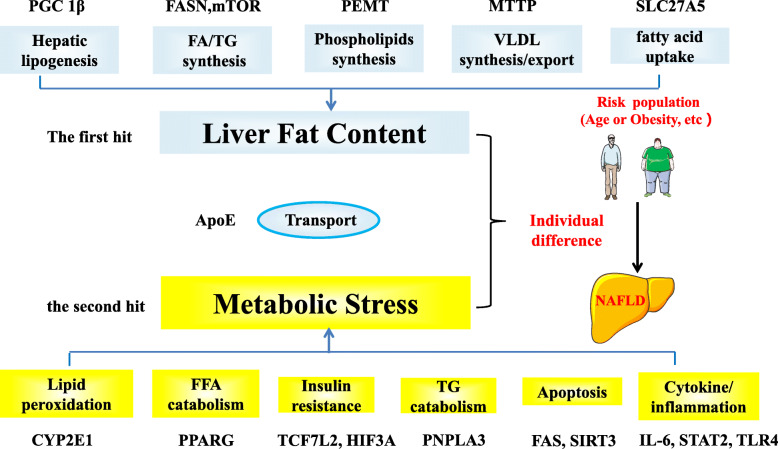
Individual susceptibility genes were selected based on the idea of “two-hit theory” affecting individual susceptibility risk of NAFLD. Among them, 7 genes (MTTP, PEMT, FASN, PGC1β, ApoE, mTOR and SLC27A5) were related to “first hit”. 10 genes (PNPLA3, Sirtuin3, CYP2E1, Fas, TLR4, TCF7L2, PPARG, IL6, STAT2 and HIF3A) were associated with “second hit”. Abbreviations: PGC1β, peroxisome proliferator-activated receptor-γ coactivator-1β; FASN, fatty acid synthase; mTOR, mechanistic target of rapamycin; PEMT, phosphatidylethanolamine N-methyltransferase; MTTP, microsomal triglyceride transfer protein; SLC27A5, soluble carrier family 27 member A5; APOE, Apolipoprotein E; CYP2E1, cytochrome P450 2E1; PPARG, Peroxisome proliferator-activated receptor-γ; TCF7L2, transcription factor 7-like 2; HIF3A, Hypoxia Inducible Factor 3 Alpha Subunit; PNPLA3, patatin-like phospholipase domain containing 3; FAS, fas cell surface death receptor; SIRT3, Sirtuin3; IL-6, interleukin-6; STAT2, signal transducer and activator of transcription 2; TLR4, toll like receptor 4

The genes affecting individual susceptibility risk for NAFLD were selected based on the “two-hit theory”. Among these, seven genes (MTTP, PEMT, FASN, PGC1β, ApoE, mTOR and SLC27A5) were associated with the “first hit”, and 10 genes (PNPLA3, Sirtuin3, CYP2E1, Fas, TLR4, TCF7L2, PPARG, IL6, STAT2 and HIF3A) were associated with the “second hit” (Fig. 2). Further screening was performed for SNPs affecting the functioning of these genes based primarily on the functional mutations identified in the ENCODE database (http://genome.ucsc.edu/ENCODE/) while also meeting a Han Chinese population minor allele frequency of > 0.05 in the 1000 Genomes database, resulting in the selection of a total of 17 candidate functional SNPs to be sent to MassARRAY for sequencing (Table 1).

**Table 1:** Allele and genotype distribution of 17 SNPs in NAFLD patients and Non-NALFD patients

Gene name	SNP	Nucleotide change	Protein change	Variant allele	Wild-type allele	*P*-value	Homozygote genotype	Heterozygote genotype	Wild-type genotype	*P*-value for HWE test (NAFLD/Non-NAFLD)	*P*-value
				NAFLD/Non -NAFLD(%)	NAFLD/Non -NAFLD(%)		NAFLD/Non -NAFLD(%)	NAFLD/Non -NAFLD(%)	NAFLD/Non -NAFLD(%)		
PPARG	rs1801282	g.12393125C > G	Pro12Ala	4.0%/3.5%	96.0%/96.5%	0.659	−/−	8.0%/7.1%	92.0%/92.9%	0.479/0.473	0.652
STAT2	rs2066811	g.56742997 T > C	p.Ile464Val	−/−	100.0%/100.0%	–	−/−	−/−	100.0%/100.0%	−/−	–
IL6	rs2069849	g.22771156C > T	p.Phe201Leu	0.4%/0.1%	99.6%/99.9%	0.401	−/−	0.7%/0.3%	99.3%/99.7%	0.953/0.980	0.401
FASN	rs2228305	g.80042792C > T	p.Val1483Ile	0.9%/0.3%	99.1%/99.7%	0.124	0.7%/−	0.4%/0.5%	98.9%/99.5%	**0..000**/0.959	0.248
MTTP	rs2306985	g.100516022C > G	p.His297Gln	67.1%/65.3%	32.9%/34.7%	0.477	44.8%/43.3%	44.8%/43.9%	10.5%/12.8%	0.810/0.526	0.657
**SIRT3**	**rs28365927**	g.236091G > A	p.Arg80Trp	14.3%/11.4%	85.7%/88.6%	0.127	1.1%/2.1%	26.4%/18.7%	72.5%/79.2%	0.178/0.126	**0.040**
mTOR	rs28990992	g.11249789G > C	p.Glu51Asp	4.4%、4.4%	95.6%/95.6%	0.963	0.7%/0.8%	7.4%/7.3%	91.9%/91.9%	**0.040**/**0.007**	0.992
Fas	rs3218619	g.90762801G > A	p.Ala16Thr	−/−	100.0%/100.0%	–	−/−	−/−	100.0%/100.0%	−/−	–
SLC27A5	rs35350976	g.59023174A > G	p.Met50Thr	5.6%/4.5%	94.4%/95.5%	0.381	0.4%/0.3%	10.5%/8.6%	89.1%/91.2%	0.883/0.787	0.682
ApoE	rs440446	g.45409167C > G	p.Asn14Lys	42.0%/39.1%	58.0%/60.9%	0.279	17.7%/14.9%	48.6%/48.4%	33.7%/36.8%	0.960/0.754	0.537
TLR4	rs4986790	g.120475302A > G	p.Asp299Gly	−/0.1%	100.0%/99.9%	0.747	−/−	−/0.3%	100.0%/99.7%	1.000/0.980	0.387
CYP2E1	rs6413419	g.135345675G > T	p.Val179Phe	−/−	100.0%/100.0%	–	−/−	−/−	100.0%/100.0%	−/−	–
PNPLA3	rs738409	g.44324727C > G	p.Ile148Met	38.5%/37.9%	61.5%/62.1%	0.827	18.4%/13.9%	40.3%/48.0%	41.3%/38.1%	**0.012**/0.695	0.097
PGC1β	rs7732671	g.149212243G > C	p.Ala203Pro	5.9%/5.0%	94.1%/95.0%	0.439	−/0.5%	11.8%/8.9%	88.2%/90.6%	0.286/0.252	0.219
TCF7L2	rs77961654	g.114925369C > A	p.Pro200Thr	26.4%/25.1%	73.6%/74.9%	0.594	8.6%/7.6%	35.7%/35.0%	55.7%/57.4%	0.172/0.173	0.870
PEMT	rs7946	g.17409560C > T	p.Val212Met	17.4%/17.6%	82.6%/82.4%	0.918	3.5%/3.2%	27.7%/28.8%	68.8%/68.0%	0.538/0.915	0.923
HIF3A	rs3764609	g.46823702A > G	p.Gln274Arg	40.0%/37.2%	60.0%/62.8%	0.287	16.8%/13.4%	46.5%/47.6%	36.7%/39.0%	0.595/0.696	0.456

*Abbreviations*: *PGC1β* peroxisome proliferator-activated receptor-γ coactivator-1β, *FASN* fatty acid synthase, *mTOR* mechanistic target of rapamycin, *PEMT* phosphatidylethanolamine N-methyltransferase, *MTTP* microsomal triglyceride transfer protein, *SLC27A5* soluble carrier family 27 member A5, *APOE* Apolipoprotein E, *CYP2E1* cytochrome P450 2E1, *PPARG* Peroxisome proliferator-activated receptor-γ, *TCF7L2* transcription factor 7-like 2, *HIF3A* Hypoxia Inducible Factor 3 Alpha Subunit, *PNPLA3* patatin-like phospholipase domain containing 3, *FAS* fas cell surface death receptor, *SIRT3* Sirtuin3, *IL-6* interleukin-6, *STAT2* signal transducer and activator of transcription 2, *TLR4* toll like receptor 4

*P*-value<0.05 considered as statistically significant (in bold)

### Statistical analysis

The odds ratios (ORs) for the candidate SNPs were first analysed by comparing 292 NAFLD patients (177 males and 114 females, mean age 48.89 ± 11.57 years) and 387 non-NAFLD patients (134 males and 251 females, mean age 41.97 ± 12.17 years). Since significant differences in gender and age were found between the two groups, an individual matching based on gender and age was performed for an OR evaluation between 223 NAFLD patients (122 males and 101 females, mean age 47.97 ± 11.01 years) and 223 non-NAFLD patients (122 males and 101 females, mean age 47.37 ± 11.41 years). Statistical analysis was performed using SPSS, version 24.0 (IBM, Chicago, IL, USA). SHEsis for Windows was used to analyse the Hardy-Weinberg equilibrium (HWE) and genotype and allele distributions between the patients and controls [17]. The baseline characteristics of the subjects were compared between the groups using a Student’s t-test if the data were normally distributed and otherwise with a Mann-Whitney U test and reported as mean ± standard deviation. Multiple linear regression analyses were used to assess the interaction between the SNPs and clinical characteristics. The association between the SNPs and NAFLD risk was estimated by computing ORs and 95% confidence intervals (CIs) from the multivariate logistic regression analyses. *P* < 0.05 was considered as statistically significant.

## Results

### Sirtuin3 rs28365927 together with gender and age were associated with a risk for NAFLD in the non-matched model

The clinical characteristics of the NAFLD and non-NAFLD patients are shown in Table 2. The NAFLD patients had a higher age, ratio of males to females, BMI, SBP, DBP, WC, HC values and serum levels of ALT, AST, fasting blood-glucose (FBG), low density lipoprotein cholesterin (LDL-c), triglyceride (TG) and total cholesterol (TC) than the non-NAFLD patients (all *P* < 0.05). In addition, serum levels of high density lipoprotein cholesterol (HDL-c), total protein (TP) and albumin in the NAFLD patients were significantly lower than in the non-NAFLD patients (*P* < 0.05). No significant differences in the other parameters were observed between the two groups of patients (all *P* > 0.05).

**Table 2:** Clinical Characteristics of Patients with NAFLD and Non-NAFLD in study population^a^

Characteristics	Non-NAFLD patients (n = 387)	NAFLD patients (n = 292)	*P* Value
Age, y	41.97 ± 12.17	48.89 ± 11.57	**0.000**
Gender,Male /Female	134/251	177/114	**0.000**
ALT, U/L	19.37 ± 14.17	33.24 ± 29.92	**0.000**
AST, U/L	22.59 ± 9.59	29.24 ± 14.47	**0.000**
FBG, mmol/L	5.11 ± 1.42	5.55 ± 1.63	**0.000**
LDL-C, mmol/L	2.81 ± 0.80	3.12 ± 0.83	**0.000**
TG, mmol/L	1.34 ± 0.92	2.51 ± 1.84	**0.000**
TC, mmol/L	4.88 ± 0.97	5.14 ± 1.07	**0.001**
HDL-C, mmol/L	1.58 ± 0.38	1.27 ± 0.33	**0.000**
BMI, kg/m^2^	22.13 ± 2.56	26.25 ± 2.55	**0.000**
SBP, mmHg	116.01 ± 17.21	123.95 ± 19.82	**0.000**
DBP, mmHg	73.43 ± 11.87	84.07 ± 15.61	**0.000**
WC, cm	73.65 ± 8.12	85.40 ± 8.29	**0.000**
HC, cm	89.62 ± 6.00	95.28 ± 6.68	**0.000**
TP,g/L	73.75 ± 3.84	72.51 ± 4.93	**0.000**
Albumin,g/L	45.97 ± 3.09	45.03 ± 4.54	**0.003**
Globulin,g/L	27.73 ± 3.38	27.88 ± 5.76	0.667
A/G	1.69 ± 0.24	1.67 ± 0.33	0.444
TBIL, umol/L	12.87 ± 8.14	12.80 ± 6.35	0.915
DBIL, umol/L	5.22 ± 2.30	5.47 ± 3.47	0.260
TBA,umol/L	3.55 ± 7.09	4.49 ± 4.98	0.232

*Abbreviations*: *BMI* Body Mass Index, *SBP* systemic blood pressure, *DBP* diastolic blood pressure, *WC* Waist circumference, *HP* Hip circumference, *TP* Total Protein, *A/G* the ratio of albumin to globulin, *TBIL* total bilirubin, *DBIL* direct bilirubin, *TBA* total bile acid, *ALT* glutamic-pyruvic transaminase, *AST* glutamic oxalacetic transaminase, *FBG* fasting blood-glucose, *LDL-C* low density lipoprotein cholesterin, *TG* triglyceride, *TC* total cholesterol, *HDL-C* high density lipoprotein cholesterol, *NAFLD* nonalcoholic fatty liver disease

^a^Values are expressed as mean ± SD or counts and compared by Student’s t-test if the data is normally distributed, otherwise Mann-Whitney U test is used, except for gender that p value stands for statistical significance using Chi-square test. *P*-value<0.05 considered as statistically significant (in bold)

The allele and genotype distributions of the 17 SNPs in the NAFLD and non-NAFLD groups demonstrate that only the genotype distributions of Sirtuin3 rs28365927 showed significant differences between the two groups (*P* < 0.05) and the genotype distributions of Sirtuin3 rs28365927 were in accordance with the HWE in both the NAFLD and non-NAFLD patients (all *P* > 0.05) (Tables 1 and 3).

**Table 3:** Allele and genotype distribution of SIRT3 rs28365927 in NAFLD patients and Non-NALFD patients

	Variant Allele(A)	Wild-type allele(G)	*P*-value	Homozygote genotype(AA)	Heterozygote genotype(AG)	Wild-type genotype(GG)	p-value for HWE test (NAFLD/Non-NAFLD)	*P*-value	Call rate(%)
	NAFLD/Non -NAFLD(%)	NAFLD/Non -NAFLD(%)		NAFLD/Non -NAFLD(%)	NAFLD/Non -NAFLD(%)	NAFLD/Non -NAFLD(%)			
unmatched	14.3%/11.4%	85.7%/88.6%	0.127	1.1%/2.1%	26.4%/18.7%	72.5%/79.2%	0.178/0.126	**0.040**	98%
matched	14.3%/9.9%	85.7%/90.1%	**0.045**	1.4%/1.4%	25.8%/17.0%	72.8%/81.7%	0.428/0.503	0.079	98%

*P*-value<0.05 considered as statistically significant (in bold)

Based on an analysis using the four genetic models, which includes dominant, recessive, additive and genotypic models, the correlations between NAFLD susceptibility and the genotype distributions of Sirtuin3 rs28365927 were explored further (Table 4). In the unmatched population, the Sirtuin3 rs28365927 GA + AA genotype was a significant risk factor for the development of NAFLD (OR = 1.976, 95%CI: 1.088–3.588, *P* = 0.025, after adjusting for age, gender and BMI) in the dominant model. There were no significant correlations between Sirtuin3 rs28365927 and NAFLD risk in the recessive and additive models, but there was a significant association between Sirtuin3 rs28365927 and higher NAFLD risk in the genotypic model (OR = 1.600, 95%CI: 1.061–2.415, *P* = 0.025, after adjusting for age and gender, GA vs GG).

**Table 4:** Association of rs28365927 with NAFLD based on four genetic models in the Study population^a^

	Unadjusted		Adjusted^a^		Adjusted^b^	
	OR (95% CI)	*P* Value	OR(95% CI)	*P* Value	OR(95% CI)	*P* Value
unmatched						
GA + AA vs GG	1.489(1.034–2.145)	**0.032**	1.532(1.026–2.287)	**0.037**	1.976(1.088–3.588)	**0.025**
AA vs GA + GG	0.491(0.129–1.868)	0.297	0.486(0.109–2.163)	0.343	1.077(0.123–9.433)	0.947
A vs G	1.286(0.925–1.786)	0.135	1.327(0.925–1.905)	0.124	1.555(0.909–2.661)	0.107
GG	1	**0.042**	1	0.052	1	0.204
GA	1.552(1.066–2.260)	**0.022**	1.600(1.061–2.415)	**0.025**	1.744(0.946–3.215)	0.075
AA	0.542(0.142–2.068)	0.370	0.541(0.121–2.417)	0.421	1.225(0.138–10.897)	0.855
matched						
GA + AA vs GG	1.662(1.054–2.619)	**0.029**	1.655(1.048–2.612)	**0.031**	1.838(1.011–3.341)	**0.046**
AA vs GA + GG	1.005(0.201–5.034)	0.995	1.004(0.199–5.053)	0.996	1.820(0.241–13.761)	0.562
A vs G	1.523(1.007–2.304)	**0.046**	1.516(1.001–2.295)	**0.049**	1.716(1.001–2.942)	**0.050**
GG	1	0.082	1	0.087	1	0.135
GA	1.705(1.069–2.721)	**0.025**	1.697(1.062–2.712)	**0.027**	1.822(0.987–3.363)	0.055
AA	1.127(0.224–5.662)	0.885	1.126(0.223–5.687)	0.885	2.084(0.273–15.935)	0.479

*Abbreviations*: *OR* odds ratio, *P*-value<0.05 considered as statistically significant (in bold);four logistic regression models (dominant:heterozygotes and variant homozygotes vs. wild homozygotes; recessive:variant homozygotes vs. wild homozygotes and heterozygotes; additive: variant allele vs. wild allele; genotypic: heterozygotes or variant homozygotes vs. wild homozygotes) were used to analyze the SNPs

^a^Binary logistic regression model was adjusted for age and gender

^b^Binary logistic regression model was adjusted for age,gender and body mass index

The above results indicate that age, gender and Sirtuin3 rs28365927 were all associated with NAFLD risk.

### Sirtuin3 rs28365927 is an independent risk factor for NAFLD in matched populations

Next, this study further verified the association between Sirtuin3 rs28365927 and the risk for NAFLD with age and sex matched between the two groups. There were significant differences in the allele distributions of Sirtuin3 rs28365927 between the NAFLD and non-NAFLD patients (*P* = 0.045), while the genotype distribution was not significantly different (*P* = 0.079) in the matched populations (Table 3). As shown in Table 4, in the matched populations, the Sirtuin3 rs28365927 GA + AA genotype was a significant risk factor for the development of NAFLD (OR = 1.662, 95%CI: 1.054–2.619, *P* = 0.029). After adjusting for age, gender and BMI, the risk of the Sirtuin3 rs28365927 GA + AA genotype was still marked (OR = 1.838, 95%CI: 1.011–3.341, *P* = 0.046) in the dominant model. There was no significant correlation between Sirtuin3 rs28365927 and NAFLD risk in the recessive model, but a significant association was found between Sirtuin3 rs28365927 and higher NAFLD risk in both the additive (OR = 1.716, 95%CI: 1.001–2.942, *P* = 0.050, after adjusting for age, gender and BMI) and genotypic models (OR = 1.697, 95%CI: 1.062–2.712, *P* = 0.027, after adjusting for age and gender, GA vs GG).

These data indicate that Sirtuin3 rs28365927 is an independent risk factor for NAFLD that is not associated with age or gender. Individuals carrying an A allele may have significantly increased NAFLD susceptibility in matched populations.

### Sirtuin3 rs28365927 affects the clinical parameters in NAFLD patients

**Fig. 3:**
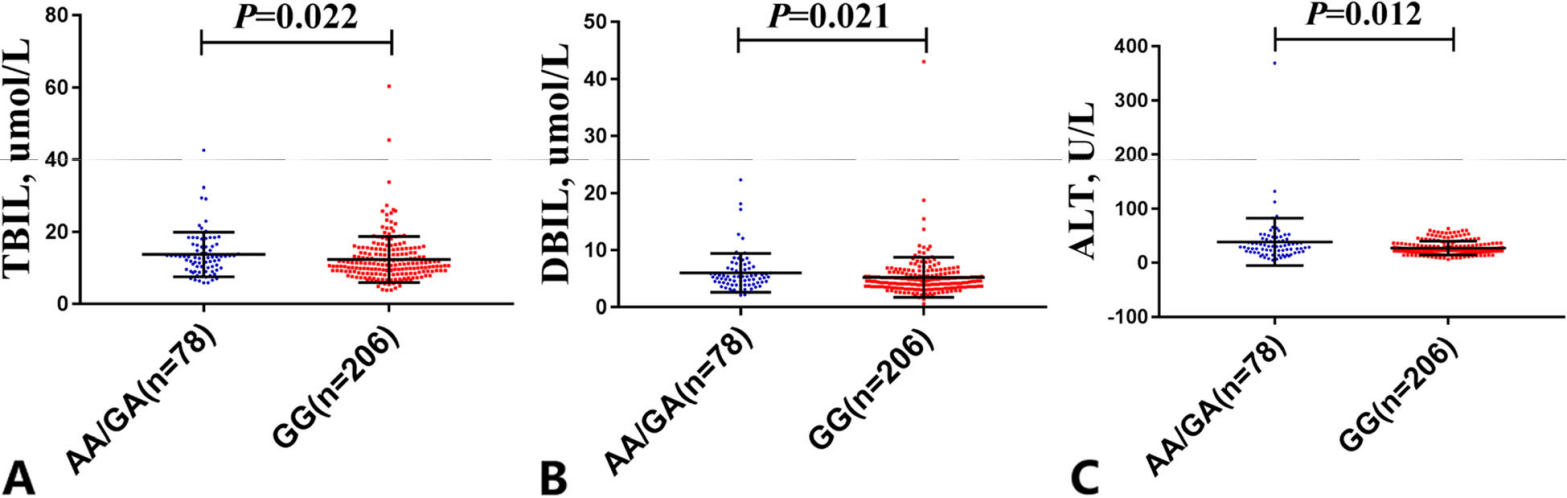
Comparison of Clinical Characteristics of SIRT3 rs28365927 genotype in NAFLD patients. **A**, Comparison of TBIL values between the SIRT3 rs28365927 AA/GA and GG genotype groups. **B**, Comparison of DBIL values between the SIRT3 rs28365927 AA/GA and GG genotype groups. **C**, Comparison of ALT values between the SIRT3 rs28365927 AA/GA and GG genotype groups. Values are expressed as mean ± SD and compared by Student’s t-test if the data is normally distributed, otherwise Mann-Whitney U test is used, except for gender that *p* value stands for statistical significance using Chi-square test. *P*-value<0.05 considered as statistically significant (in bold). Abbreviations: NAFLD, non-alcoholic fatty liver disease; SIRT3, Sirtuin3; TBIL, total bilirubin; DBIL, direct bilirubin; ALT, glutamic-pyruvic transaminase

**Fig. 4:**
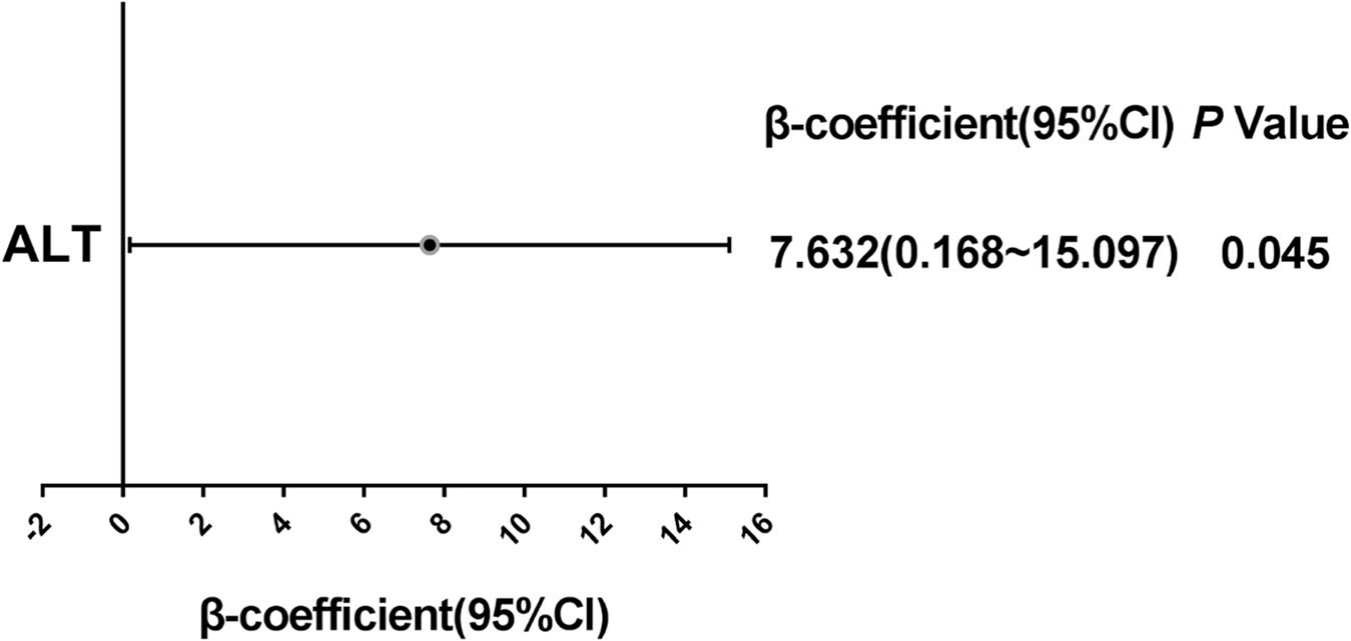
Significant associations between rs28365927 A Carriers (AA+AG) and ALT in NAFLD patients in a covariate adjusted linear regression analysis. β-coefficient: estimated quantitative effect of rs28365927 A Carriers on a phenotype of NAFLD, *P*: level of statistical significance of the covariate (age and gender) adjusted linear regression analysis. *P*-value<0.05 considered as statistically significant (in bold). Abbreviations: NAFLD, non-alcoholic fatty liver disease; SIRT3, Sirtuin3; ALT, glutamic-pyruvic transaminase

To investigate the potential correlation, this study compared the Sirtuin3 rs28365927 genotype with the clinical parameters of the NAFLD patients. As shown in Table 5 and Fig. 3, among the NAFLD patients, serum levels of TBIL, DBIL and ALT in rs28365927 A allele carriers (GA + AA) were 11.1, 14.7 and 41.5% higher, respectively, than in non-carriers (GG) (*P* = 0.022, *P* = 0.021, *P* = 0.012, respectively). In a covariate adjusted linear regression analysis, as shown in Table 6 and Fig. 4, rs28365927 A carriers (AA + AG) were positively correlated with higher ALT among the NAFLD patients (*P* = 0.045).

**Table 5:** Clinical Characteristics of SIRT3 rs28365927 A Carriers(AA+AG) and Non-Carriers(GG) in the NAFLD Population^a^

Characteristic	Carriers (*n* = 78)	Non-Carriers (*n* = 206)	*P* Value
Age, y	49.38 ± 11.89	48.78 ± 11.47	0.834
Gender,Male /Female	48/30	123/82	0.813
BMI, kg/m^2^	25.80 ± 2.78	26.40 ± 2.47	0.123
SBP, mmHg	121.72 ± 18.15	124.45 ± 20.35	0.322
DBP, mmHg	82.28 ± 14.85	84.73 ± 15.96	0.623
WC, cm	85.02 ± 8.70	85.64 ± 8.21	0.747
HP, cm	94.43 ± 6.29	95.65 ± 6.88	0.220
TP,g/L	72.48 ± 5.33	72.42 ± 4.81	0.434
Albumin,g/L	45.00 ± 4.49	45.00 ± 4.63	0.529
Globulin,g/L	27.54 ± 3.08	27.72 ± 5.34	0.636
A/G	1.70 ± 0.50	1.66 ± 0.25	0.765
TBIL, umol/L	13.74 ± 6.20	12.37 ± 6.40	**0.022**
DBIL, umol/L	6.01 ± 3.41	5.24 ± 3.52	**0.021**
TBA,umol/L	4.66 ± 5.13	4.45 ± 5.00	0.959
ALT, U/L	38.64 ± 43.94	27.31 ± 12.92	**0.012**
AST, U/L	30.82 ± 14.47	28.62 ± 14.66	0.250
FBG, mmol/L	5.54 ± 1.41	5.56 ± 1.74	0.389
LDL-C, mmol/L	3.04 ± 0.80	3.160.86	0.314
TG, mmol/L	2.17 ± 0.96	2.66 ± 2.10	0.318
TC, mmol/L	5.11 ± 0.89	5.17 ± 1.13	0.968
HDL-C, mmol/L	1.26 ± 0.30	1.26 ± 0.33	0.779

*Abbreviations*: *BMI* Body Mass Index, *SBP* systemic blood pressure, *DBP* diastolic blood pressure, *WC* Waist circumference, *HP* Hip circumference, *TP* Total Protein, *A/G* the ratio of albumin to globulin, *TBIL* total bilirubin, *DBIL* direct bilirubin, *TBA* total bile acid, *ALT* glutamic-pyruvic transaminase, *AST* glutamic oxalacetic transaminase, *FBG* fasting blood-glucose, *LDL-C* low density lipoprotein cholesterin, *TG* triglyceride, *TC* total cholesterol, *HDL-C* high density lipoprotein cholesterol, *NAFLD* nonalcoholic fatty liver disease

^a^Values are expressed as mean ± SD and compared by Student’s t-test if the data is normally distributed, otherwise Mann-Whitney U test is used, except for gender that p value stands for statistical significance using Chi-square test. *P*-value<0.05 considered as statistically significant (in bold)

**Table 6:** Significant associations between rs28365927 A Carriers(AA+AG) and Clinical Characteristics in a covariate adjusted linear regression analysis in the NAFLD Population^a^

Characteristic	β-coefficient	STAT	*P* Value
BMI, kg/m^2^	−0.569	−1.506	0.133
SBP, mmHg	−3.082	−1.117	0.265
DBP, mmHg	−2.583	−1.196	0.233
WC, cm	−0.440	−0.373	0.709
HP, cm	−1.148	−1.196	0.233
TP,g/L	0.116	0.185	0.853
Albumin,g/L	0.060	0.106	0.915
Globulin,g/L	−0.424	−0.558	0.577
A/G	0.039	0.897	0.370
TBIL, umol/L	1.220	1.511	0.132
DBIL, umol/L	0.703	1.542	0.124
TBA,umol/L	0.154	0.156	0.876
ALT, U/L	7.632	2.013	**0.045**
AST, U/L	2.446	0.857	0.393
FBG, mmol/L	0.025	0.130	0.897
LDL-C, mmol/L	−0.101	−0.903	0.367
TG, mmol/L	−0.437	−1.968	0.050
TC, mmol/L	−0.019	−0.135	0.893
HDL-C, mmol/L	−0.003	−0.080	0.936

*Abbreviations*: *BMI* Body Mass Index, *SBP* systemic blood pressure, *DBP* diastolic blood pressure, *WC* Waist circumference, *HP* Hip circumference, *TP* Total Protein, *A/G* the ratio of albumin to globulin, *TBIL* total bilirubin, *DBIL* direct bilirubin, *TBA* total bile acid, *ALT* glutamic-pyruvic transaminase, *AST* glutamic oxalacetic transaminase, *FBG* fasting blood-glucose, *LDL-C* low density lipoprotein cholesterin, *TG* triglyceride, *TC* total cholesterol, *HDL-C* high density lipoprotein cholesterol, *NAFLD* nonalcoholic fatty liver disease

^a^β-coefficient: estimated quantitative effect of rs28365927 A Carriers on a phenotype of NAFLD, STAT: T statistic, *P*: level of statistical significance of the covariate(age and gender) adjusted linear regression analysis. *P*-value<0.05 considered as statistically significant (in bold)

## Discussion

NAFLD is a disease that is affected by multifactorial risk [18], among which genetic variation is one of the important factors in individual susceptibility to NAFLD [19]. For example, Microsomal triglyceride transfer protein (MTTP) rs1800591 increases the development of NALFD by affecting VLDL secretion, while Uncoupled protein 2 (UCP2) rs695366 reduces the progression of NASH through lipid antioxidant activity [20, 21]. This study have newly discovered that Sirtuin3 rs28365927 is significantly associated with NAFLD risk, regardless of whether it was in a matched group or not. In the unmatched groups, this study found that the Sirtuin3 rs28365927 GA + AA genotype was a significant risk factor for the development of NAFLD in the dominant model, and there was a significant association between Sirtuin3 rs28365927 and higher NAFLD risk in the genotypic model (GA vs GG). In the matched groups, this study also found the Sirtuin3 rs28365927 GA + AA genotype was a significant risk factor for the development of NAFLD in the dominant model. There was a significant association between Sirtuin3 rs28365927 and higher NAFLD risk in both the additive and genotypic models (GA vs GG). In contrast, no risk association was discovered for PNPLA3 and other SNP loci, which may be mainly due to different populations. All of the subjects included in this study were of Han Chinese origin, and this study conclusions may not be applicable to other ethnicities.

Sirtuins are an evolutionarily conserved NAD^+^-dependent deacetylase family, and there are seven of them in mammals. Three (Sirtuin3, Sirtuin4 and Sirtuin5) are primarily localized in mitochondria [22]. Sirtuin3 is the only sirtuin that has robust deacetylase activity in mitochondria, and it is a crucial gatekeeper of redox status, the epigenetic landscape and lipid homeostasis in hepatocytes [23, 24]. In most cases, increased Sirtuin3 expression is protective from NAFLD. The introduction of Sirtuin3 protects liver function, reduces liver fibrosis, reduces inflammatory response and prevents hepatocyte apoptosis, and the overexpression of Sirtuin3 protects hepatocytes from mitochondrial apoptosis by promoting the mitosis required by Bnip 3 [25]. The flavonoid dihydromyricetin can help prevent NAFLD by improving mitochondrial respiration and liver cell redox homeostasis by increasing Sirtuin3 expression [22]. The overexpression of Sirtuin3 in MTP^+/−^ mice significantly reduced the acetylation of MTP as compared with β-galactosidase controls and increased mitochondrial fatty acid oxidation and reduced hepatic steatosis, CD68 and serum ALT levels [26]. Studies have shown that theacrine can inhibit liver steatosis and liver inflammation in NAFLD by promoting the metabolism of acylcarnitine through Sirtuin3/LCAD signalling pathways [27]. In mice, Berberine reduced the NAFLD induced by a high-fat diet by activating Sirtuin3 [28]. Protocatechuic acid has a protective effect on NAFLD by increasing Sirtuin3 expression [29]. Interestingly, Sirtuin3 overexpression makes the liver and hepatocytes susceptible to palmitate-induced cell death [30]. So far, no studies have reported the relationship between Sirtuin3 genetic variation and NAFLD. Sirtuin3 rs28365927 is located in chr11:236091(GRCH38.p13), belonging to the Arg80Trp missense variant according to PUBMED database. Sirtuin3 rs28365927 may alter the binding motif of PAX-5 and Znf143 transcription factors by using HaploReg 4.1 database. In this study, Sirtuin3 rs28365927 was found to be significantly associated with NALFD susceptibility and among the NAFLD patients, serum levels of TBIL, DBIL and ALT in rs28365927 A allele carriers (GA + AA) were 11.1, 14.7 and 41.5% higher, respectively, than in non-carriers (GG) (Fig. 3). In addition, among the NAFLD patients, Rs28365927 A carriers (AA + AG) were positively correlated with higher ALT (Fig. 4). These results indicate that the Sirtuin3 rs28365927 A allele may be associated with an impairment of liver function.

**Fig. 5:**
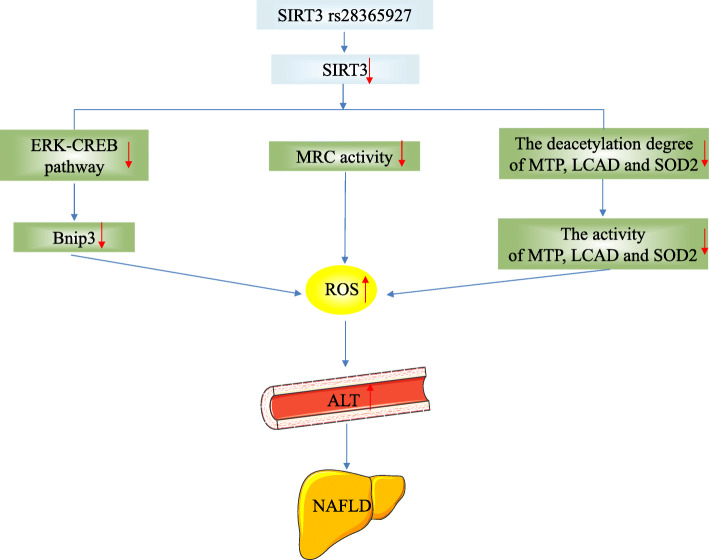
The potential mechanism pathway of SIRT3 rs28365927 to increase the risk of NAFLD. Sirtuin3 rs28365927 may leads to the production of reactive oxygen species (ROS) by decreasing the activity of MRC, inactivating the ERK-CREB pathway and reducing the activity of MTP/LCAD/SOD2, and the production of ROS leads to the death of liver cells, which in turn leads to an increase in serum ALT levels [2, 23–26, 34–37]. Abbreviations: SIRT3, Sirtuin3; MRC,mitochondrial respiratory chain; ROS, reactive oxygen species; ERK,extracellular signal regulated kinase; CREB,the cAMP response element binding protein; Bnip3,BCL2 interacting protein 3; MTP,mitochondrial trifunctional protein; LCAD,long chain acyl coa dehydrogenase; SOD2,superoxide dismutase 2; NAFLD,Non-alcoholic fatty liver disease

ALT level is one of the high risk indexes for NAFLD, and Sirtuin3 rs28365927 may contribute to the risk for NAFLD through an effect on ALT level. NAFLD has been strongly associated with ALT activity in previous studies [31–33]. Mild ALT elevation is largely attributed to NAFLD [34, 35]. Sirtuin3 rs28365927 may leads to the production of reactive oxygen species (ROS) by decreasing the activity of mitochondrial respiratory chain (MRC), inactivating the extracellular signal regulated kinase-cAMP response element binding protein (ERK-CREB) pathway and reducing the activity of mitochondrial trifunctional protein (MTP)/ long chain acyl coa dehydrogenase (LCAD)/superoxide dismutase 2 (SOD2), and the production of ROS leads to the death of liver cells, which in turn leads to an increase in serum ALT levels (Fig. 5, [2, 25–28, 36–39]. However, the potential mechanism of Sirtuin3 rs28365927 in increasing NAFLD susceptibility requires further study.

## Study strength and limitations

This study is the first comprehensive case-controlled study to research the relationship between genetic variants of the Sirtuin3 gene and susceptibility to NAFLD, and these findings are important because they provide a new point for exploring the role that Sirtuin3 plays in NAFLD based on genetic factors. Sirtuin3 rs28365927 is a newly discovered independent risk factor SNP associated with NAFLD in the Han Chinese population. The limitation of this study is that ultrasound is used in the diagnosis of NAFLD, which is not an accurate means to diagnose fatty liver and can only be seen when the pathological changes are relatively obvious. The study population was limited to the Han Chinese population and may not be applicable to other ethnic groups.

## Conclusion

Sirtuin3 rs28365927 functional variant confers to the high risk of non-alcoholic fatty liver disease in Chinese Han population. The rs28365927 A allele significantly increased the ALT levels of NAFLD patients. As more and more genetic polymorphisms are reported, it may be possible in the future to individualize treatment based on the individual risk and disease progression of NAFLD patients.

## Supplementary Information

**Additional file 1: Table S1.** Clinical Characteristics of SIRT3 rs28365927 A Carriers and Non-Carriers in the Study Population^a^.

## Data Availability

None.

## Abbreviations

PGC1β: Peroxisome proliferator-activated receptor-γ coactivator-1β
FASN: Fatty acid synthase
mTOR: Mechanistic target of rapamycin
PEMT: Phosphatidylethanolamine N-methyltransferase
MTTP: Microsomal triglyceride transfer protein
SLC27A5: Soluble carrier family 27 member A5
APOE: Apolipoprotein E
CYP2E1: Cytochrome P450 2E1
PPARG: Peroxisome proliferator-activated receptor-γ
TCF7L2: Transcription factor 7-like 2
HIF3A: Hypoxia Inducible Factor 3 Alpha Subunit
PNPLA3: Patatin-like phospholipase domain containing 3
FAS: Fas cell surface death receptor
SIRT3: Sirtuin3
IL-6: Interleukin-6
STAT2: Signal transducer and activator of transcription 2
TLR4: Toll like receptor 4
BMI: Body Mass Index
SBP: Systemic blood pressure
DBP: Diastolic blood pressure
WC: Waist circumference
HP: Hip circumference
TP: Total Protein
A/G: The ratio of albumin to globulin
TBIL: Total bilirubin
DBIL: Direct bilirubin,
TBA: Total bile acid
ALT: Glutamic-pyruvic transaminase
AST: Glutamic oxalacetic transaminase
FBG: Fasting blood-glucose,
LDL-C: Low density lipoprotein cholesterin,
TG: Triglyceride
TC: Total cholesterol
HDL-C: High density lipoprotein cholesterol
NAFLD: Nonalcoholic fatty liver disease
