# Supplementary material for: Sirtuin3 rs28365927 functional variant confers to the high risk of non-alcoholic fatty liver disease in Chinese Han population
Source: Lipids Health Dis. 2021 Aug 26;20:92. doi: 10.1186/s12944-021-01520-x (PMC8390275; doi:10.1186/s12944-021-01520-x)
Supplement: Supplementary file 1 — Additional file 1: Table S1. Clinical Characteristics of SIRT3 rs28365927 A Carriers and Non-Carriers in the Study Populationa. [file 12944_2021_1520_MOESM1_ESM.docx]

# *Additional files*

**Sirtuin3 rs28365927 functional mutation confers to the high risk of non-alcoholic fatty liver disease in Chinese Han population**

**Li-jie Chen**^a,b,c,d^, Jing Guo^a,b,c,d^, Song-xia Zhang^a,b,c,d^, Ying Xu^a,b,c,d^, Qing Zhao ^a,b,c,d^, Wei Zhang^a,b,c,d^, Jian Xiao^e^, **Yao Chen**^a,b,c,d*^

*^a^**Department of Clinical Pharmacology, Xiangya Hospital,* *Central South University, Changsha, Hunan, China.*

*^b^Institute of Clinical Pharmacology, Central South University, Changsha, Hunan, China.*

*^c^Engineering Research Center of Applied Technology of Pharmacogenomics, Ministry of Education, Changsha, Hunan, China.*

*^d^National Clinical Research Center for Geriatric Disorders, Changsha, Hunan, China.*

*^e^Department of Pharmacy, Xiangya Hospital, Central South University, Changsha, Hunan, China.*

***Corresponding author:** Associate Prof. Yao Chen, Department of Clinical Pharmacology, Xiangya Hospital, Central South University, Changsha, 410008, P.R. China. Phone: +86-731-8480-5380, Email: cbohua@csu.edu.cn

**Other authors’ emails:**

Li-jie Chen: [cljzndx@163.com](mailto:cljzndx@163.com)

Jing Guo: 1437312590@qq.com

Song-xia Zhang: zsx5201218@163.com

Ying Xu: [xying15@csu.edu.cn](mailto:xying15@csu.edu.cn)

Qing Zhao: 1184684198@qq.com

Wei Zhang:yjsd2003@163.com

Jian Xiao: [admanoas@163.com](mailto:admanoas@163.com)

**Other information for the paper:**

Pages: 43 Pages

Figures: 5 Figures

Tables: 6 Tables

References: 37 References

Word counts: abstract:247; text:2428

**Table S1.** Clinical Characteristics of SIRT3 rs28365927 A Carriers and Non-Carriers in the Study Population^a^

| Characteristic | Overall Series |  |  |  | NAFLD Patients |  |  |  | Non-NAFLD patients | | |
| --- | --- | --- | --- | --- | --- | --- | --- | --- | --- | --- | --- |
|  | Carriers  (n=157) | Non-Carriers  (n= 507) | P Value |  | Carriers  (n= 78) | Non-Carriers  (n= 206) | P Value |  | Carriers  (n= 79) | Non-Carriers  (n= 301) | P Value |
| Age, y | 45.66±13.39 | 44.73±12.11 | 0.492 |  | 49.38±11.89 | 48.78±11.47 | 0.834 |  | 41.97±13.84 | 42.00±11.78 | 0.898 |
| Gender,Male /Female | 75/82 | 228/276 | 0.578 |  | 48/30 | 123/82 | 0.813 |  | 27/52 | 105/194 | 0.876 |
| BMI, kg/m^2^ | 23.91±3.16 | 23.68±3.29 | 0.493 |  | 25.80±2.78 | 26.40±2.47 | 0.123 |  | 22.35±2.56 | 22.07±2.57 | 0.407 |
| SBP, mmHg | 119.96±18.22 | 119.20±18.89 | 0.385 |  | 121.72±18.15 | 124.45±20.35 | 0.322 |  | 118.22±18.24 | 115.68±16.99 | 0.246 |
| DBP, mmHg | 77.82±13.65 | 78.01±14.86 | 0.930 |  | 82.28±14.85 | 84.73±15.96 | 0.623 |  | 73.42±10.74 | 73.53±12.20 | 0.843 |
| WC, cm | 78.70±10.00 | 78.14±10.01 | 0.511 |  | 85.02±8.70 | 85.64±8.21 | 0.747 |  | 73.44±7.75 | 73.70±8.18 | 0.918 |
| HP, cm | 91.83±6.59 | 91.84±6.97 | 0.930 |  | 94.43±6.29 | 95.65±6.88 | 0.220 |  | 89.67±6.05 | 89.58±5.98 | 0.968 |
| TP,g/L | 73.35±4.74 | 73.16±4.29 | 0.386 |  | 72.48±5.33 | 72.42±4.81 | 0.434 |  | 74.21±3.93 | 73.66±3.83 | 0.385 |
| Albumin,g/L | 45.46±3.81 | 45.58±3.84 | 0.928 |  | 45.00±4.49 | 45.00±4.63 | 0.529 |  | 45.92±2.96 | 45.98±3.14 | 0.915 |
| Globulin,g/L | 27.88±3.39 | 27.67±4.24 | 0.223 |  | 27.54±3.08 | 27.72±5.34 | 0.636 |  | 28.23±3.66 | 27.64±3.31 | 0.176 |
| A/G | 1.68±0.40 | 1.68±0.24 | 0.148 |  | 1.70±0.50 | 1.66±0.25 | 0.765 |  | 1.66±0.27 | 1.69±0.23 | 0.114 |
| TBIL, umol/L | 12.63±5.61 | 12.86±7.94 | 0.508 |  | 13.74±6.20 | 12.37±6.40 | **0.022** |  | 11.53±4.75 | 13.20±8.82 | 0.194 |
| DBIL, umol/L | 5.42±2.79 | 5.26±2.88 | 0.397 |  | 6.01±3.41 | 5.24±3.52 | **0.021** |  | 4.83±1.85 | 5.28±2.36 | 0.294 |
| TBA,umol/L | 5.24±10.13 | 3.69±3.89 | 0.705 |  | 4.66±5.13 | 4.45±5.00 | 0.959 |  | 6.13±14.96 | 2.86±1.80 | 0.685 |
| ALT, U/L | 28.65±33.26 | 24.42±19.51 | 0.080 |  | 38.64±43.94 | 27.31±12.92 | **0.012** |  | 18.79±10.36 | 19.61±15.13 | 0.806 |
| AST, U/L | 27.12±12.63 | 25.89±13.05 | 0.234 |  | 30.82±14.47 | 28.62±14.66 | 0.250 |  | 21.72±6.44 | 22.93±10.35 | 0.871 |
| FBG, mmol/L | 5.30±1.09 | 5.31±1.66 | 0.150 |  | 5.54±1.41 | 5.56±1.74 | 0.389 |  | 5.06±0.59 | 5.13±1.59 | 0.573 |
| LDL-C , mmol/L | 2.94±0.87 | 2.95±0.82 | 0.971 |  | 3.04±0.80 | 3.160.86 | 0.314 |  | 2.84±0.93 | 2.80±0.77 | 0.990 |
| TG, mmol/L | 1.72±0.90 | 1.88±1.66 | 0.367 |  | 2.17±0.96 | 2.66±2.10 | 0.318 |  | 1.29±0.58 | 1.35±1.00 | 0.558 |
| TC, mmol/L | 5.02±0.96 | 4.99±1.04 | 0.374 |  | 5.11±0.89 | 5.17±1.13 | 0.968 |  | 4.94±1.03 | 4.87±0.96 | 0.626 |
| HDL-C , mmol/L | 1.42±0.35 | 1.46±0.40 | 0.309 |  | 1.26±0.30 | 1.26±0.33 | 0.779 |  | 1.57±0.32 | 1.59±0.39 | 0.853 |

Abbreviations :BMI Body Mass Index,SBP systemic blood pressure,DBP diastolic blood pressure,WC Waist circumference,HP Hip circumference,TP Total Protein, A/G the ratio of albumin to globulin, TBIL total bilirubin, DBIL direct bilirubin,TBA total bile acid,ALT glutamic-pyruvic transaminase,AST glutamic oxalacetic transaminase, FBG fasting blood-glucose, LDL-C low density lipoprotein cholesterin, TG triglyceride, TC total cholesterol, HDL-C high density lipoprotein cholesterol,NAFLD nonalcoholic fatty liver disease.

^a^Values are expressed as mean±SD and compared by Student’s t-test if the data is normally distributed, otherwise Mann-Whitney U test is used, except for gender that p value stands for statistical significance using Chi-square test. *P*-value＜0.05 considered as statistically significant (in bold).
